# A systematic review of pharmaceutical price mark-up practice and its implementation

**DOI:** 10.1016/j.rcsop.2021.100020

**Published:** 2021-05-06

**Authors:** Kah Seng Lee, Yaman Walid Kassab, Nur Akmar Taha, Zainol Akbar Zainal

**Affiliations:** a Faculty of Pharmacy, University of Cyberjaya, Cyberjaya, Selangor, Malaysia; b Faculty of Pharmacy, Universiti Teknologi MARA, Puncak Alam, Selangor, Malaysia

**Keywords:** Prescription fees, Healthcare costs, Drug costs, Cost control, Pharmaceutical economics, Price list

## Abstract

Pharmaceutical products, apart from being essential for medical treatment, are of high value and heavily regulated to ensure the prices are controlled. This systematic review was conducted to identify pharmaceutical pricing mark-up control measures, specifically in the wholesale and retail sectors. The search method comprised the following databases: PubMed, Science Direct, Springer Link, ProQuest, and EBSCOhost and Google Scholar. The results were filtered systematically from the inception of the aforementioned databases until 23 April 2021. Eligible studies were those focusing on the implementation of pharmaceutical pricing strategies that involve a) mark-ups of medicine, and b) pharmaceutical cost control measures. A total of 13 studies were included in this review: seven covered European countries, four covered Asian countries, one covered the USA and one covered Canada. The main points of discussion in the qualitative synthesis were the implementation of medicine mark-ups, price mark-up regulatory strategies and the outcomes of these regulatory strategies. Our findings suggest that Western countries have a lower mark-up margin, around 4% to 25% of the original purchased price, compared to Asian countries, up to 50%.

## 1 Introduction

The World Health Organization (WHO) defines a mark-up as the difference between the purchase price (cost price) and the retail price of a product.^1^ A mark-up is the sum of all the additional charges and costs imposed on a product in order to recover production costs and generate a profit. In a product's distribution cycle, commercial practices which involve discounts, rebates and other trade promotions, as well as wholesale regulation and retail remuneration, could result in flexible mark-ups.^2 3^ For example, medicines which are originally higher in cost could be marked up lower to create incentives, thus making a differential impact on innovator brand and generic drugs at varied price ranges.^4^ Mark-ups may be represented as a single defined value, or as a percentage of purchase price, or as a combination of said defined value and purchase price.^5^^,^^6^ The mark-up ratio can be calculated by dividing the difference between the selling price and the purchase price by the purchase price. It has been well established that both community pharmacies and hospitals could charge mark-ups, and mark-ups could be charged on both prescription drugs and over-the-counter medicine which may or may not be covered by health insurance schemes.^7^

Unregulated pharmaceutical prices in private healthcare settings have become a major source of consumer complaints and lead to unnecessary inflation of medicine prices.^8^ This free pricing policy has resulted in pharmaceutical price disparity among general practitioner clinics and private hospitals.^9^ Even though a legal and policy framework of medicine price regulation and control are in place for most countries, the detailed pricing mechanism and the current medicine prices, including the private negotiation schemes, are usually not disclosed to the general public.^10^ This also complicates the estimation of international reference pricing among different countries.^11^ Weakening economies due to the onslaught of the COVID-19 pandemic has strengthened the need to scrutinize negotiations of mark-up margins for both the private and public sectors. On the same note, as the medicine expenditure continues to increase over the years, there is a pressing need for governments to implement cross-border collaboration to improve access to medicine^11^ as well as cost control measures to ensure the affordability and accessibility of medicines.^12^^,^^13^

The current body of knowledge mainly focuses on the pharmaceutical pricing in specific regions or by specific treaties (e.g. Europe, Organization for Economic Co-operation and Development); by drug category (anticancer drugs, vaccines, innovator or generic drugs) or by income groups (low, middle income countries).^14^, ^15^, ^16^, ^17^, ^18^ Closer examination of these reviews shows that they do not present particular effects for measures taken to control pharmaceutical pricing in individual countries. They mainly discuss the general application of a price control measure. Although they provide a clear picture of how a policy works, they do not research the effect and suitability of the said policy. Our review fills this gap in the literature in the latest update on the price disparity and medicine mark-ups among countries with different cost control measures. It could be useful for policymakers when devising an appropriate policy for medicine price control,^19^^,^^20^ especially for the practice of regressive mark-ups whereby lower mark-ups on the initially high-priced products can have a significant effect on the sale of originator brand and generic medicines as they are sold at different market prices.^21^^,^^22^

This systematic review was conducted to identify pharmaceutical mark-up control measures, specifically in the wholesale and retail sectors. The purpose of specifying the scope at the wholesaler and retailer levels is because these two actors hold the most weight when setting a selling price of a drug. Furthermore, we also aimed to compare and examine the pharmaceutical mark-up situation in various countries. A comparison of the policy and scheme structures between countries could be used by policymakers and researchers to identify similarities and contrasts in comparable structures embedded within the pharmaceutical pricing schematic framework and policies.

## 2 Methods

The current systematic review was conducted on the basis of the Cochrane guidelines for systematic reviews of health promotion and public health.^23^ The search was done from the inception of the databases up to 23 April 2021. The following databases were utilised as the search tools: PubMed, Science Direct, Springer Link, ProQuest, and EBSCOhost. A grey literature search was conducted through official government publications, World Health Organization/Health Action International (WHO/HAI) reports, and Google Scholar. Abstracts, conference proceedings and studies which were written in languages other than English were excluded, as were articles which consisted of only abstracts.

### 2.1 Search strategy and inclusion criteria

The exact electronic search strategy is presented in Supplementary Information 1. Several keywords and medical subject headings (MeSHs), including health expenditures, drug costs, drug utilization, and cost control, were used to identify the scope of inclusions. The keywords were individually used or combined using Boolean logic, whichever was applicable, as the search terms in the databases mentioned above.

The inclusion criteria applied were studies aiming to determine or examine pharmaceutical price mark-ups, cost control measures and health-related expenditures. The studies, apart from fulfilling the stipulated aims, also had to contain (a) outcome measures including method of calculating mark-ups, and (b) measures used for medicine cost control.

The exclusion criteria were non-English articles and non-full text articles (abstract only), letters to editors, short communication, and opinion articles. We also excluded articles in pre-print form.

**Fig. 1:**
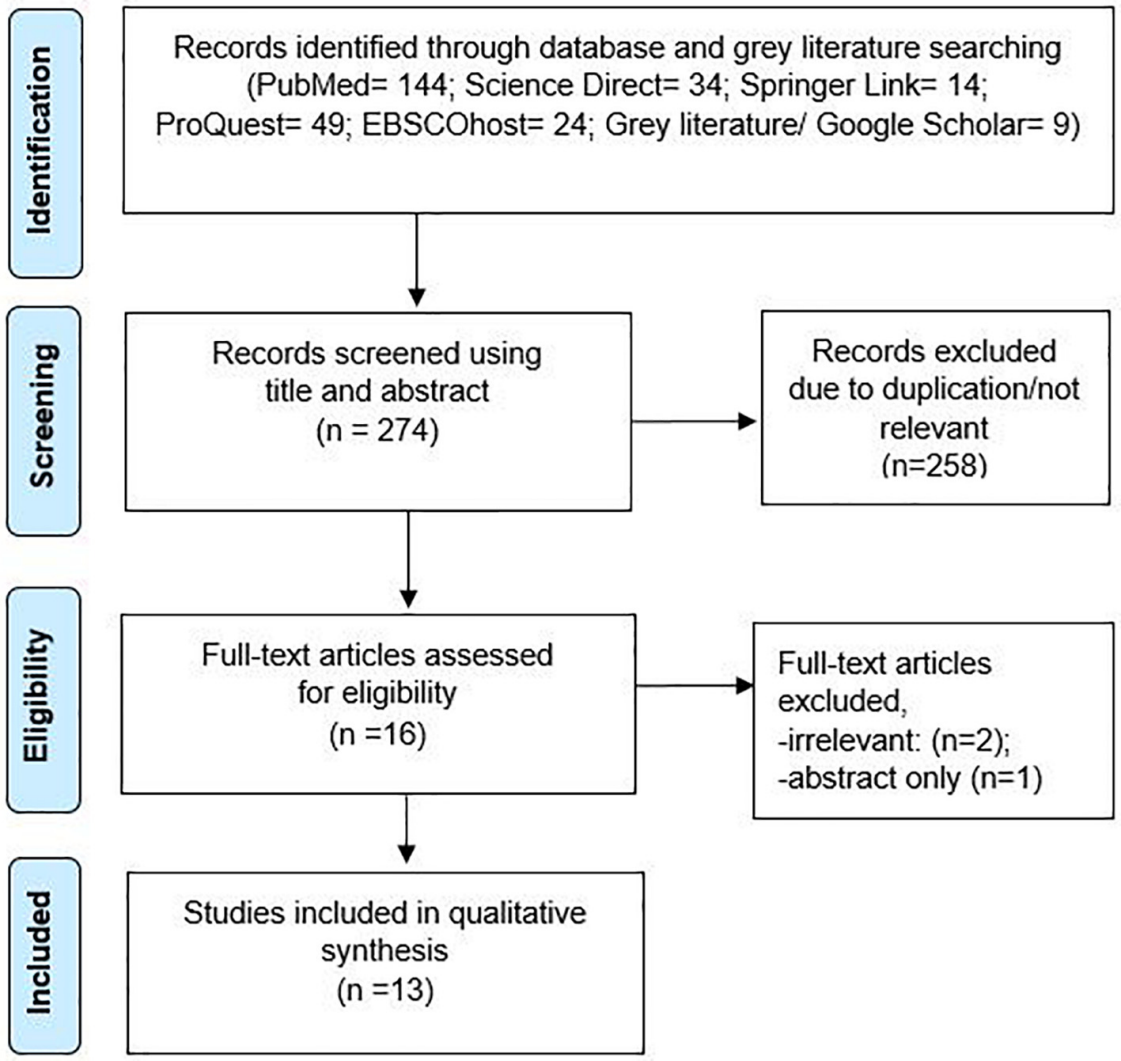
PRISMA diagram demonstrating the search strategy and its results.

The Preferred Reporting Items for Systematic Reviews and Meta-Analyses (PRISMA) method and flow diagram were used to illustrate the workflow of the systematic review (Fig. 1). After obtaining relevant articles using the search terms, duplicates were removed. Irrelevant content was filtered out through title and abstract read-throughs. Following the exclusion process, the remaining studies were screened based on the full texts' eligibility. Suitable articles were closely screened by two reviewers (K.S.L. and Y.W.K.) for eligibility. Where discrepancy arose, it was resolved through discussion and where necessary, the third reviewer (Z.A.Z.) decided upon any dispute. After conducting the full text screening, the results were compared to identify similarities and contrasts. The complete PRISMA checklist can be found in Supplementary Information 2.

### 2.2 Data extraction

A data tabulation form was prepared for effective data extraction. The form was used to tabulate source of publication, year of publication, country of study, and related price control policy. The data extraction form for all identified articles was prepared by both aforementioned reviewers, and the results were then compared to achieve inter-reviewer agreement. Further evaluation of the extracted data was conducted by grouping the countries according to their region, i.e. Asian and Western countries. In addition, mark-ups strategies and their outcomes were identified. All the policies and strategies were divided into public and private sectors. A conceptual framework proposed by the Irish Economic and Social Research Institute was used as a guide to decipher and examine the included studies.^24^ In this particular report, policies that involved pharmaceutical supply chains and the links therein—for instance importers, manufacturers, wholesalers, pharmacists and general practitioners—including demand and supply of pharmaceuticals (manufacturer, wholesaler, retailer pricing), reimbursement and delivery systems were reviewed. Since most of the returned results related to pharmaceutical policy studies were case descriptions, we did not proceed with a risk of bias assessment.

## 3 Results

### 3.1 Search results and included studies

The initial search came up with 274 results, of which 258 were excluded due to duplication or irrelevance. At the screening stage, 16 articles underwent an eligibility assessment. Two articles were not relevant and one was a conference proceeding that only had an abstract section, so the final results comprised 13 studies.

### 3.2 Data extraction

#### 3.2.1 Study general characteristics

Among the retrieved studies, two covered multiple countries: Espin, et al.^3^ covering the USA, Italy and France, and Vandoros^25^ covering Germany and Norway. Four studies covered Asian countries: China, Japan, Korea and Taiwan. The remaining studies focused on the UK, Greece, Sweden and Canada, respectively. Table 1 shows the main characteristics of the included studies. The articles generally revolved around three main points: the implementation of medicine mark-ups (*n* = 8); price mark-up regulatory strategies (*n* = 12); and outcomes of the regulatory strategies (*n* = 13). The setting of price mark-ups mainly focused on wholesalers (*n* = 4) and retailers (*n* = 6). The included studies depicted the mark-up policies in the public sector (*n* = 5), the private sector (n = 5), or the juxtaposition in both sectors (n = 6).

**Table 1:** General characteristics of the included studies.

No.	Author/Reference	Country/Location	Study design/report	Legislative
1	Burstall (1997)	UK	Health policy guideline	Pharmaceutical Price Regulation Scheme (PPRS)
2	Ljungkvist et al. (1997)	Sweden	Health policy guideline	Medical Products Agency
3	Dong et al. (1999)	China	Health policy guideline	The Drug Administration Law at the mainland China
4	Morgan (2000)	Canada	Empirical research (PhD project)	National Prescription Drug Utilization Information System (NPDUIS)
5	Iizuka (2001)	Japan	Empirical Essay	Yakka Kijyun (reimbursement tariff list)
6	Lee et al. (2006)	Taiwan	Quasi-experimental design	Taiwanese National Health Insurance (NHI)
7	Vandoros (2010)	Germany	Empirical research (PhD project)	Federal Institute for Pharmaceuticals and Medical Devices
8	Hågå (2002)	Norway	Empirical research	Act on Medicinal Products article 6 and chapter 12 of the appurtenant Regulation on Medicinal Products (Norwegian law)
9	Kastanioti et al. (2016)	Greece	Analytic review	Structural reforms in the healthcare system and other provisions
10	Espin et al. (2018)	USA	Health policy guideline	Medicare
11	Folino-Gallo (2008)	Italy	Pricing guideline	Italian Medicines Agency (AIFA)
12	Grandfils (2008)	France	Health policy guideline	Comité Economique du Médicament (CEM)
13	Suh et al. (2018)	Korea	Empirical research	National Health Insurance (NHI)

#### 3.2.2 Pricing policy

Table 2 illustrates the different pharmaceutical pricing policies reported in the included studies. Two countries (China and Norway) included both wholesale and retail mark-ups in the public and private sectors. For both public and private sectors, Japan mentioned mark-ups solely in retail while for the USA, a single policy was addressed, which is public wholesale mark-ups.

**Table 2:** Pharmaceutical pricing policies characteristics.

No.	References	Country/Location	Public wholesale mark-ups	Public retail mark-ups	Private wholesale mark-ups	Private retail mark-ups
1	Burstall (1997)	UK	25%	√	25%	√
2	Ljungkvist et al. (1997)	Sweden	√	X	√	4.2–8.1%
3	Dong et al. (1999)	China	10–15%	15–30%	10–15%	15–30%
4	Morgan (2000)	Canada	√	√	√	√
5	Iizuka (2001)	Japan	X	10%	X	10%
6	Lee et al. (2006)	Taiwan	√	5–40%	√	5–40%
7	Vandoros (2010)	Germany	X	√	X	√
8	Hågå (2002)	Norway	5–7%	5–8%	5–7%	5–8%
9	Kastanioti et al. (2016)	Greece	√	√	√	√
10	Espin et al. (2018)	USA	17%	X	X	X
11	Folino-Gallo (2008)	Italy	√	√	√	√
12	Grandfils (2008)	France	√	√	√	√
13	Suh et al. (2018)	Korea	√	53.55%	√	53.55%

√: mark-ups were practiced but the margin of mark-ups was not reported; X: not practiced.

#### 3.2.3 Qualitative synthesis

It should be noted that while the studies showed the comparison in term of price control strategies, the outcomes were not assessed in a similar manner, which ruled out a further quantitative comparison. Western countries that practise mark-ups have shown a lower mark-up margin (4% to 25% of the original purchased price) compared to Asian countries (up to 50%). The summaries of the qualitative synthesis of the included studies are presented in Table 3.

**Table 3:** Summaries of the qualitative synthesis of the included studies.

Refe	Reference	Dispensing fee	Wholesale mark-up regulatory strategies					Retail mark-up regulatory strategies					
			Fixed fee	Regressive fixed fee	Fixed %	Regressive %	Cap	Fixed fee	Regressive fixed fee	Fixed %	Regressive %	Cap	Dispensing fee
1	Burstall (1997)	–	–	–	√	–	–	–	–	√	–	Yes. 21%	
2	Ljungkvist et al. (1997)	–	–	–	√	–	No	–	–	–	√	No	No
3	Dong et al. (1999	–	–	–	√	–	–	–	–	√	–	No	No
4	Morgan (2000	–	–	–	√	–	No	–	–	√	–	No	No
5	Iizuka (2001)	–	–	–	–	–	No	–	–	√	–	No	No
6	Lee et al. (2006)	<10%	√	–	–	–	No	√	–	–	–	No	No
7	Vandoros (2010	–	–	–	–	–	No	–	–	–	√	Yes	No
8	Hågå (2002)	–	–	–	√	–	–	–	–	√	–	No	No
9	Kastanioti et al. (2016)	–	–	–	√	–	No	–	–	√	–	No	No
10	Espin et al. (2018)	–	–	–	–	–	–	–	–	–	–	No	No
11	Folino-Gallo (2008)	–	–	–	√	–	No	√	√	–	–	No	No
12	Grandfils (2008)	–	–	–	–	√	No	–	–	–	√	No	No
13	Suh et al. (2018)	–	–	–	√	–	No	–	–	√	–	No	No

The Asian countries included in the studies were China, Japan, Korea and Taiwan. All four countries allow price mark-ups at the retail level. In Korea, public and private retail mark-ups can go up as high as 53.55% above the wholesale price. The mark-up percentage in China ranges from 15% to 30%, while in Taiwan the range falls between 5% and 40%. The price mark-up in Japan is fixed at 10%, in both public and private retail.

In terms of national health coverage through national health insurance, Japan spearheaded the single-payer system back in 1958 with the passing of the national health insurance law at the federal level. Korea and Taiwan followed suit with nationwide universal health coverage in 1989 and 1995, respectively. Only Taiwan imposes a dispensing fee on top of the retail price, but this is less than 10% of the final selling price. Over the years, the Taiwanese government has recommended generic grouping, which is the referencing of the price scheme based on a drug's chemical equivalence and the reduction of flat payment. Flat payment was a rate introduced in 1995 to allow private clinics to profit from drug sales. This led to a 9.6% pharmaceutical expenditure reduction in Taiwan in 2003, whereby generic grouping and flat drug payment rate reduction accounted for approximately USD 35.5 million and USD 359.3 million cumulative savings, respectively.^26^

In Japan, there is no price cap or fixed percentage in terms of mark-ups for wholesalers. For retailers, however, there is a 10% fixed percentage. The Japanese government practises ‘yakka kijyun’, a pricing formula which acts as the benchmark of prescription drug prices. The regulation was revised in 1992 and has been in force ever since. To obtain the average wholesale price of a product, the government carries out extensive surveys. The results are used as the standard reference in order to regulate the retail price. According to an official government report, ‘yakka kijyun’ lowered prescription drug costs by 8.2% between 1992 and 1997.^27^

Similar to Japan, Korea has imposed a fixed percentage for retail mark-up, only with a much higher percentage, namely 53.55%. However, the Korean government has had a drug pricing system in place since 2006. The pricing system ensures that when drug patents expire, the prices of innovator drugs are reduced to 80% of the initial prices. Typically, the price of generic drugs are fixed at 90% of the off-patent price and might differ when the generics are marketed. In such cases, earlier entry of the generics to the market could secure a higher price. In 2012, the Korean government introduced a single price system to make sure generics are sold at 85% of the off-patent price. From one year after the patent expiry, the drug can be sold at 53.55% of the original price (when the patent was still in force). A single price system has successfully reduced drug costs in targeted pharmaceuticals, particularly in antidiabetics. The expenses on overall antidiabetics and reduced price antidiabetics fell by 6% and 23%, respectively, in the year after the new pricing system was implemented.^28^

China allows both wholesale and retail mark-ups in the public and private sectors. The wholesaler can mark up the drug price around 10–15%. The wholesale price is controlled by the public service pricing bureau under the provision of the Central Pricing Commission of China. Mark-ups are permissible from the provincial wholesale to national wholesale levels. Generally, the range of the mark-ups is approximately 10% to 15%. Thereafter, retailers can further increase the price by 15–30% of the wholesale price. The mark-ups also differ according to the origin of the drugs. Modern drugs can be marked up by 15%, while Chinese herbal preparation drugs can be marked up by 16%. For raw Chinese herbs, the mark-up is 30%. Hospitals and general practitioners are allowed to earn mark-up percentages of 15% and 30% for Western drugs and Chinese medicines, respectively. Moreover, the mark-ups can be pushed up to 40% if bulk purchase discounts plus bonus stock are taken into consideration. The mark-ups across every level of the supply chain allow the service providers to make profits. As a result, the medicine prices are often skewed in comparison with the manufacturer and the retailer costs.^29^

Our findings show that China has applied a price mark-up policy involving almost every level of the supply chain. On average, the mark-ups at each level can range from 10% to 30%. This makes China the most heavily marked-up of the four studied Asian countries in terms of drug pricing at the retail level. Although Taiwan had a reported mark-up margin of at most 40% for drugs, and Korea of almost 54%, these are the mark-up margins for the entire supply chain, not just the retailers. In Asia, labour is plentiful and inexpensive because of the huge population. For example, China and India combined account for approximately 36% of the world's population. Furthermore, the raw materials are more easily sourced in these countries. When workers and materials are abundant, the production cost decreases. Also, middle-class and upper-class Asians have greater spending power now as compared to last decades due to globalisation and the country's strong economic growth. Overall, the price and profit margin increase due to greater buying power.

In Western countries, drug price mark-ups are also widely practiced across the supply chain. For example, in Italy, France, Greece, and Canada, no price cap or additional dispensing fee is imposed. In Italy, pharmaceutical coverage is provided for residents under the national health service.^30^ A price regulatory strategy fixes the mark-up percentage at the retail level at 22.9%. Meanwhile at the wholesale level, the government regulates the wholesale price by putting a fixed 7.3% mark-up percentage. Under the national health service regulation, statutory discounts by retailers are allowed in order to create regressive margins.^9^

The situation is slightly different in France, where both wholesaler and retailer prices are controlled by a regressive percentage margin.^3^ Wholesalers can place mark-ups of 10.74% on the manufacturer price, or increase the selling price by 9.7% of the pharmacy purchase price. However for the latter they are taxed at 1.2%. Retailers are advised not to substitute innovator drugs with generics, as generics are entitled to lower mark-ups.^31^ Similarly, in Canada and Greece, the wholesalers are restricted by a fixed margin, but the retailers are not tied to any format of fixed or regressive fixed fee.^32^^,^^33^ In Canada, drug prices are regulated by federal and provincial governments. Mark-up policies were introduced under the National Prescription Drug Utilization Information System plan. Pharmaceutical price mark-ups are applied in all the provinces across Canada, except Manitoba. The provincial government of Manitoba instead utilizes actual acquisition costs to let retailers bill the drug procurement cost plus a wholesaler mark-up. As a result, generics are priced 7% to 9% higher in Manitoba than elsewhere in Canada.^32^

In Greece, the introduction of pharmaceutical mark-ups, centralized public procurement of pharmaceuticals, the increased use of generics, and rises in household co-payments have been implemented to minimize pharmaceutical spending on both the supply and demand sides. The mark-up scheme, generally imposed on wholesaler and pharmacies, differs with drug types: reimbursable, over-the-counter list, and negative list.^33^ As a result, major price reductions and enhanced co-payments have been observed with a substantial 34.5% reduction in the total current health expenditurebetween 2009 and 2015.^34^

In Norway, public and private sector drug pricing is regulated by a fixed mark-up percentage. For wholesalers, the margin is within 5% to 7%, while retailers can adjust their pricing within 5% to 8% of the original price.^25^ The Norwegian Medicines Agency uses the Guideline on Pricing of Medicinal Products to decide on the mark-up margin. The implementation of a price index on innovator drugs has helped to increase the market share of generics to some extent, thus promoting price competition by reducing the domination of innovator drugs. Indirectly, the mark-up policy has stimulated the sale of generics.^35^

In the USA, more strategies are applied to prevent drug over-pricing. Innovator drug producers are given discounts and rebates which indirectly reduce production expenditures by approximately 18% annually. Between 2010 and 2014, the amount of discount was increased to 24% of the total cost of original brand drugs. On top of that, wholesalers are allowed to put mark-ups of up to 17% on the wholesale price.^3^

Likewise, the UK practises mark-up percentage control, with a 25% margin imposed on the wholesale level. In addition, retailer mark-ups are capped at 21% of the initial selling price. The UK government has taken a different approach in controlling drug pricing. Instead of regulating the price of innovator drugs directly, it controls the rate of return on capital in sales. Furthermore, the government introduced the Pharmaceutical Price Regulation Scheme (PPRS) in 1993. The PPRS is a mechanism used by the UK Department of Health to ensure that the National Health System (NHS) obtains high-quality brand-name medicines at a reasonable price. This includes a non-contractual agreement between the Department of Health and the Association of the British Pharmaceutical Industry (ABPI). The plan applies to all brand-approved drugs on the NHS.^36^ PPRS involves a cross-industry cap on sales growth whereby cash rebates are paid by drug companies to the NHS every quarter. In a five-year period, the rate of return on capital is around 17% to 21%.^36^

In Sweden, there are no regulatory measures to control the price at the manufacturer's level, which means drug producers are free to decide on the selling price. In January 1993, the Swedish government introduced a reference price system. This system works by setting a reference price, which is 110% of the lowest price among a group of drugs with similar package sizes. To measure the lowest price, about 1000 packages of different drugs are grouped in 50 groups of similar package sizes. If the cost of procurement exceeds the reference price, the balance is non-reimbursable. At the retail level, the Swedish government imposes regressive mark-ups of between 4.2% and 8.1% in the private sector.^37^

The German government introduced the Medicines Price Ordinance, which functions as a guideline to control wholesalers' maximum mark-up margins. Following that, retailers are permitted to mark up the price on top of the wholesaler's price, though the percentage mark-up is fixed by law. In Germany, wholesalers practise a common culture whereby they offer discounts to retailers as a way of securing long-term contracts. In 1993 and 1994, a price suspension endorsed by the government allowed the price freeze of drugs which did not fall under the reference pricing category. The retail price of prescription drugs was fixed at 95% of the price sold in 1992; the price of non-prescription medicines was controlled at 98% of the selling price in the previous year for both 1993 and 1994. Prices for new drugs marketed between the middle and end of 1992 were retroactively fixed at a range of 95% to 98%.^25^ Following that, as new drug could only be priced at a maximum of 98% from its wholesaler price, the usage of generics had increased.^25^

## 4 Discussion

Our current review examines the schematic and regulatory framework of pharmaceutical distribution and mark-ups in the identified countries. Technically, the medicine price is considered as fair if it is affordable to the patient while covering the retailer's costs plus a reasonable profit margin. Contradictorily, from the patient's point of view, a drug price is considered as “fair” when it is affordable, sustainable, and value for money. Perhaps it is contentious to determine the accepted profit margin of a medicine price. This is because fairness in drug prices does not solely reflect the benefit of buyers. Fairness means the policy is advantageous towards the patients too. A fair price should be inclusive of manufacturing costs, research and development costs, licensing costs, and a reasonable amount of profit. This is known as the price floor. The price ceiling is determined by these factors. A fair price which benefits both parties should be within the range of price floor and price ceiling. If a policy places the drug pricing under the price floor, it is undeniable that drug manufacturers and sellers would be forced to delay the production of drugs. Likewise, if a policy favours the sellers and sets drug prices above the ceiling, people would not be able to afford them, thus jeopardizing the balance of supply and demand.

Different countries adopt their own methods of pharmaceutical market management. Some countries employ various medical and pharmaceutical policies to balance the incurred healthcare costs and income generated from mark-ups. Others, like Italy, Norway and France, provide subsidies or do not charge for medication in public healthcare facilities. Most countries have implemented price control mechanisms as recommended by the WHO, such as external reference pricing which is commonly used by most European countries to determine the mark-up margin.^38^^,^^39^ The external reference pricing uses the price of a pharmaceutical product in one or several countries to derive a benchmark or reference price in order to set or negotiate the price of the product in the host country.^2^ Such a mechanism is not without its drawbacks. First, pricing estimation using external reference pricing will be inaccurate if the market intelligence collected the wrong medicine pricing details, including in terms of strength, dosage size, pack size and active ingredients.^38^^,^^39^ Second, setting a low price for a medicine measured using external referencing pricing could potentially lead to a medicine going out of stock in a particular country simply because the pharmaceutical companies will tend to divert supply to neighbouring countries that offer a better price.^40^

Our findings indicate that the majority of studies on drug pricing mark-ups have been conducted in European countries. In fact, there is a lack of pharmaceutical price control especially in developing countries, for example Chile, Ghana and Somalia.^41^ The absence of price control policies leads to unregulated selling price. Although the price of drugs may be cheaper in such regions compared to Europe and the USA, the quality of drugs might be compromised.^42^^,^^43^ Furthermore, it is difficult to compare drug-pricing mark-ups among different countries, since not all of them are applying mark-up controls consistently across all type of medicines. A clearer picture will be presented if more studies focusing on medicine mark-ups are done according to the drug pharmacological grouping.

Among the nine Western countries examined, only the UK imposed a price cap system, which controlled the maximum retail mark-ups at 21% of the wholesale price. Italy was the sole country where fixed fees and regressive fixed fees were regulated at the retailer level.^30^ In general, price mark-ups across the pharmaceutical supply chain in Western countries fall within the range of 4% to 25%, which is almost 50% lower than Asian countries. This may be a consequence of the countries' varying political stances, financial situations, and pharmaceutical regulations.^44^^,^^45^ Most Europeans are protected by a national medical scheme or health insurance.^46^ The reason behind these measures might be that the original price of the drug is already high.^47^ Many pharmaceutical companies manufacture their products in Asia, due to the cheaper labour costs and easier access to raw materials. It seems prudent to propose that an import cost is should be added on top of the original drug price, making it difficult to raise the mark-up ceiling level in Western countries.

The advent of effective and reliable biologics and precision medicines are taking the pharmaceutical industry a big step forward. But new, highly individualized drugs are meaningless if most patients are unable to afford them. Similarly, there is no point in pumping funds into pharmaceutical research and development when the investors are unable to sustain the pharmaceutical lifecycle management. Hence, every country should have a price control policy to protect the lives of patients, and the livelihood of pharmaceutical industry players.^48^

With price regulation, patients are able to afford medications which in most cases are extremely important to keep them healthy.^49^ In the USA, it is often being cited that prescription medications are more expensive than in other countries in the region. It is estimated that around 30% of patients in the USA are unable to afford their prescriptions, and later succumb to their illnesses.^50^^,^^51^ Since drugs are essential to healthcare, some companies are taking advantage of their blockbuster drugs that monopolize the market. With price control measures in place, this situation could be avoided. However, controlling the selling price of medicine might lead to price fluctuation in other parts of the pharmaceutical supply chain. For example, drug utilization tends to increase if the price of a drug is decreased tremendously. On the contrary, in tandem with the price drop, unfavourable marketing could lead to less demand and subsequent rationing. The equilibrium of drug supply and demand might be at risk due to the manufacturers' unwillingness to produce the required volume of drugs. In most cases, pharmaceutical companies rely on high profit margins of drug sales to sustain research and development. For instance, the leading pharma companies have drastically slashed budgets for antibiotic innovation due to an unfavourable return of investment caused by the fast development of antibiotic resistance.^52^^,^^53^

With respect to the advantages and disadvantages of drug pricing mark-up controls, it is important for a country's policymakers to study and evaluate the economic impact of having a mark-up policy. As discussed earlier, different countries have their own health financing and reimbursement schemes which suit their needs at that particular period of time.^45^ Nevertheless, deciding on the “perfect” price control strategy poses an enormous challenge, so authorities should do extensive research when drafting new or revising existing regulations.

## 5 Limitations and recommendations

One of the main limitations of this review is that the percentage mark-ups are difficult to interpret because of unstandardized interpretation and calculation methods. Due to patent protection, most innovator products are not locally produced whereas generic drugs could be mass-produced domestically. Therefore, foreign data might not be accessible to the researchers. Furthermore, the dimension or universality of public insurance programs can vary by country, and the scale of private insurance programs may vary accordingly. These variables could influence the purchase price, which is integral to the mark-up margin estimation. Next, some data in the included articles might be outdated because policy and regulation could have changed in recent years. Our included studies were dated from 1997 to 2018; perhaps some regulations have since been revised or are no longer applicable. In order to prevent misjudgements, the discussion presented in this review has assimilated the most recent systematic reviews and white papers. Furthermore, the quoted mark-up margins serve as a snapshot of pricing at a specific time point, which reflects certain policies or policy changes in that particular country.

Seemingly it is hard to strike a perfect balance between strict regulation which results in lower margins and flexible mark-ups which result in higher usage. We propose three main recommendations for future studies:

1) Research that focuses on a specific region rather than worldwide in order to better identify the factors affecting drug pricing.
2) More in-depth research aiming at policy reformation and adoption should be conducted to analyse the success of a price-setting policy.
3) A comprehensive exploration should be carried out into how countries vary in terms of the public and private insurance markets, the manufacturing or import of pharmaceuticals, and related non-pharmaceutical legislation that may be aimed at controlling overall healthcare costs.

## 6 Conclusion

Aspects such as the implementation of medicine mark-ups, price mark-up regulatory strategies and outcomes of the regulatory strategies are commonly discussed in the included studies. Based on the findings, it is prudent to suggest that Western countries have a lower mark-up margin, around 4% to 25% of the original purchased price, compared to Asian countries, where it is up to 50%. Our results reveal the dissimilarities of medicine mark-up schemes in term of medicine pricing policy, geographic location and economics of the country. In general, developed and developing countries eagerly implement pricing policies to control pharmaceutical-related expenditures. Based on the systematic review, these policies typically involve price mark-up control measures to achieve their goals. The major impact of these control measures is improved affordability. By managing the drug prices, pharmaceutical expenditures can be optimised. This review could serve as a useful reference for health regulatory agencies in drafting cost control measures because it provided a detailed review of implementation of the price control policies, procedures and price mark-up control measures with regard to its success and challenges.

## Funding

This research received no external funding.

## Declaration of Competing Interest

The authors declare no conflict of interest.

## Appendix A Supplementary data

Supplementary Information 1: Search strategy Image 1

Supplementary Information 2: PRISMA Checklist Image 2
