# Supplementary material for: A systematic review of pharmaceutical price mark-up practice and its implementation
Source: Explor Res Clin Soc Pharm. 2021 May 6;2:100020. doi: 10.1016/j.rcsop.2021.100020 (PMC9031039; doi:10.1016/j.rcsop.2021.100020)
Supplement: Supplementary file 1 — Supplementary Information 1: Search strategy [file mmc1.pdf]

Article Title: A Ssystematic Rreview of Ppharmaceutical price Mmark-up Ppractice and Iits Iimplementation

Journal: Exploratory Research in Clinical and Social Pharmacy
Article number: 100020
https://doi.org/10.1016/j.rcsop.2021.100020

Supplementary Information 1: Search strategy

Concept 1<br>Health expenditure/cost of medicine	(‘health expenditure’ [MeSH Terms] OR ‘expenditures’ [MeSH Terms] OR ‘cost* [MeSH Terms]) AND ('medicine' OR ‘drug’ [MeSH Terms])
Concept 2<br>Measures	‘cost control’ [MeSH Terms] OR ‘Mark-up’ [MeSH Terms] OR ‘Fixed fee’ [MeSH Terms] OR ‘Regressive fixed fee’ [MeSH Terms] OR ‘Fixed Percentage’ [MeSH Terms] OR ‘Regressive Percentage’ [MeSH Terms] OR ‘Cap Mark-up’ [MeSH Terms] OR ‘Dispensing fee’ [MeSH Terms])
Concept 3	(‘economics, pharmaceutical’ [MeSH Terms]) OR ‘pharmaceutical’ [MeSH Terms]
