# Supplementary material for: A systematic review of pharmaceutical price mark-up practice and its implementation
Source: Explor Res Clin Soc Pharm. 2021 May 6;2:100020. doi: 10.1016/j.rcsop.2021.100020 (PMC9031039; doi:10.1016/j.rcsop.2021.100020)
Supplement: Supplementary file 2 — Supplementary Information 2: PRISMA Checklist [file mmc2.pdf]

PRISMA 2009 Checklist

Section/topic	#	Checklist item	Reported on page #
**TITLE**			
Title	1	A Systematic Review of Pharmaceutical Price Mark-up Practice and Its Implementation	1
**ABSTRACT**			
Structured summary	2	Pharmaceutical products, apart from being essential for medical treatment, are of high value and heavily regulated to ensure the prices are controlled. This systematic review was conducted to identify pharmaceutical pricing mark-up control measures, specifically in the wholesale and retail sectors. The search method comprised the following databases: PubMed, Science Direct, Springer Link, ProQuest, and EBSCOhost and Google Scholar. The results were filtered systematically from the inception of the aforementioned databases until 23 April 2021. Eligible studies were those focusing on the implementation of pharmaceutical pricing strategies that involve a) mark-ups of medicine, and b) pharmaceutical cost control measures. A total of 13 studies were included in this review: seven covered European countries, four covered Asian countries, one covered the USA and one covered Canada. The main points of discussion in the qualitative synthesis were the implementation of medicine mark-ups, price mark-up regulatory strategies and the outcomes of these regulatory strategies. Our findings suggest that Western countries have a lower mark-up margin, around 4% to 25% of the original purchased price, compared to Asian countries, up to 50%.	1
**INTRODUCTION**			
Rationale	3	Unregulated pharmaceutical prices in private healthcare settings have become a major source of consumer complaints and lead to unnecessary inflation of medicine prices.[1] This free pricing policy has resulted in pharmaceutical price disparity among general practitioner clinics and private hospitals.[2] The current body of knowledge mainly focuses on the pharmaceutical pricing in specific regions or by specific treaties (e.g. Europe, Organisation for Economic Co-operation and Development); by drug category (anticancer drugs, vaccines, innovator or generic drugs) or by income groups (low, middle income countries).[3-7] Our review fills this gap in the literature in the latest update on the price disparity and medicine mark-ups among countries with different cost control measures. It could be useful for policymakers when devising an appropriate policy for medicine price control,[8, 9] especially for the practice of regressive mark-ups whereby lower mark-ups on the initially high-priced products can have a significant effect on the sale of originator brand and generic medicines as they are sold at different market prices.[10, 11]	2, 3
Objectives	4	This systematic review was conducted to identify pharmaceutical mark-up control measures, specifically in the wholesale and retail sectors. The purpose of specifying the scope at the wholesaler and retailer levels is because these two actors hold the most weight when setting a selling price of a drug. Furthermore, we also aimed to compare and examine the pharmaceutical mark-up situation in various countries.	3

PRISMA 2009 Checklist

**METHODS**			
Protocol and registration	5	The study protocol for this systematic review has been registered and published in the international prospective register of systematic reviews (PROSPERO) with the registration number.	N/A
Eligibility criteria	6	The inclusion criteria applied were studies aiming to determine or examine pharmaceutical price mark-ups, cost control measures and health-related expenditures. The studies, apart from fulfilling the stipulated aims, also had to contain (a) outcome measures including method of calculating mark-ups, and (b) measures used for medicine cost control. The exclusion criteria were non-English articles and non-full text articles (abstract only), letters to editors, short communication, and opinion articles. We also excluded articles in pre-print form.	4,5
Information sources	7	The search was done from the inception of the databases up to 23 April 2021 using PubMed, Science Direct, Springer Link, ProQuest, and EBSCOhost and Google Scholar.	5
Search	8	**Please see Table 1.** Search Strings Used	5
Study selection	9	Identification, title and abstract screening, full text eligibility assessment, included in systematic review.	4
Data collection process	10	The literature was collected using PubMed, Science Direct, Springer Link, ProQuest, and EBSCOhost and Google Scholar databases.	
Data items	11	The literature relating to pharmaceutical, or drug, or medicine mark-up and cost control measures till April 2021 was included in this study	
Risk of bias in individual studies	12	It was not performed in this study because most of the returned results related to pharmaceutical policy studies in developing countries are case descriptions, so quality and risk of bias assessments could not be performed on them	
Summary measures	13	Not applicable because we do not perform meta-analysis for this study	
Synthesis of results	14	Not applicable because we do not perform meta-analysis for this study	

PRISMA 2009 Checklist

Section/topic	#	Checklist item	Reported on page #
Risk of bias across studies	15	Not applicable because we do not perform meta-analysis for this study	
Additional analyses	16	Not applicable because we do not perform meta-analysis for this study	
**RESULTS**			
Study selection	17	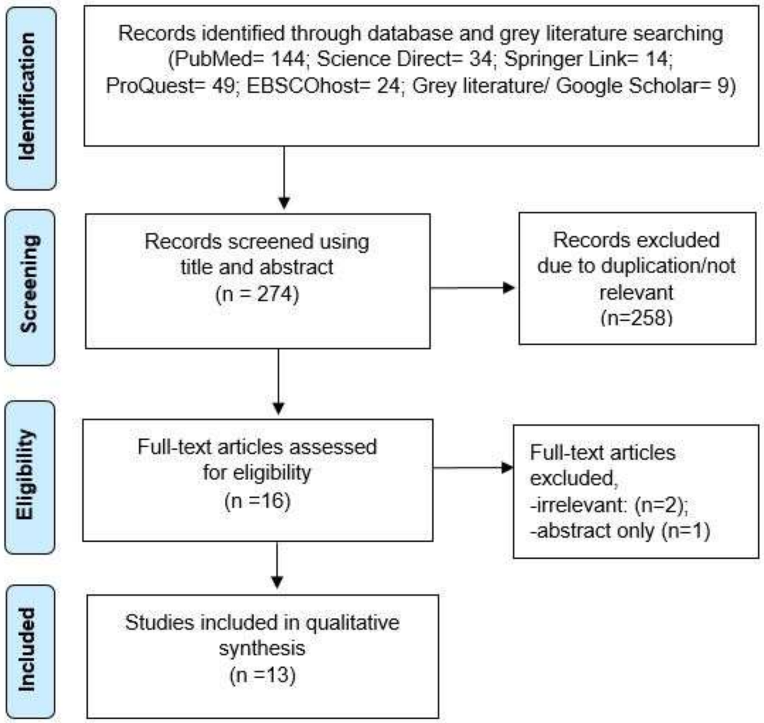	4

Study characteristics	18	Refer to Supporting Information **S1.** Characteristics of the Included Studies and **S2.** Summary of Outcomes of Included Studies	S1, S2
Risk of bias within studies	19	Not applicable	
Results of individual studies	20	Not applicable	
Synthesis of results	21	Not applicable	
Risk of bias across studies	22	Not applicable	
Additional analysis	23	Not applicable	
**DISCUSSION**			
Summary of evidence	24	Different countries adopt their own methods of pharmaceutical market management. Some countries employ various medical and pharmaceutical policies to balance the incurred healthcare costs and income generated from mark-ups. Others, like Italy, Norway and France, provide subsidies or do not charge for medication in public healthcare facilities. Most countries have implemented price control mechanisms as recommended by the WHO, such as external reference pricing which is commonly used by most European countries to determine the mark-up margin.[12, 13] The external reference pricing uses the price of a pharmaceutical product in one or several countries to derive a benchmark or reference price in order to set or negotiate the price of the product in the host country.[14] Such a mechanism is not without its drawbacks. First, pricing estimation using external reference pricing will be inaccurate if the market intelligence collected the wrong medicine pricing details, including in terms of strength, dosage size, pack size and active ingredients.[12, 13] Second, setting a low price for a medicine measured using external referencing pricing could potentially lead to a medicine going out of stock in a particular country simply because the pharmaceutical companies will tend to divert supply to neighbouring countries that offer a better price.[15]<br>Our findings indicate that the majority of studies on drug pricing mark-ups have been conducted in European countries. In fact, there is a lack of pharmaceutical price control especially in developing countries, for example Chile, Ghana and Somalia.[16] The absence of price control policies leads to unregulated selling price. Although the price of drugs may be cheaper in such regions compared to Europe and the USA, the quality of drugs might be compromised.[17, 18] With respect to the advantages and disadvantages of drug pricing mark-up controls, it is important for a country's policymakers to study and evaluate the economic impact of having a mark-up policy. As discussed earlier, different countries have their own health financing and reimbursement schemes which suit their needs at that particular period of time.[19] Nevertheless, deciding on the "perfect" price control strategy poses an enormous challenge, so authorities should do extensive research when drafting new or revising existing regulations.	16, 17, 18, 19

Limitations	25	One of the main limitations of this review is that the percentage mark-ups are difficult to interpret because of unstandardized interpretation and calculation methods. Due to patent protection, most innovator products are not locally produced whereas generic drugs could be mass-produced domestically. Therefore, foreign data might not be accessible to the researchers. Furthermore, the dimension or universality of public insurance programs can vary by country, and the scale of private insurance programs may vary accordingly. These variables could influence the purchase price, which is integral to the mark-up margin estimation. Next, some data in the included articles might be outdated because policy and regulation could have changed in recent years. Our included studies were dated from 1997 to 2018; perhaps some regulations have since been revised or are no longer applicable. In order to prevent misjudgements, the discussion presented in this review has assimilated the most recent systematic reviews and white papers. Furthermore, the quoted mark-up margins serve as a snapshot of pricing at a specific time point, which reflects certain policies or policy changes in that particular country.	20
Conclusions	26	Aspects such as the implementation of medicine mark-ups, price mark-up regulatory strategies and outcomes of the regulatory strategies are commonly discussed in the included studies. Based on the findings, it is prudent to suggest that Western countries have a lower mark-up margin, around 4% to 25% of the original purchased price, compared to Asian countries, where it is up to 50%. Our results reveal the dissimilarities of medicine mark-up schemes in term of medicine pricing policy, geographic location and economics of the country. In general, developed and developing countries eagerly implement pricing policies to control pharmaceutical-related expenditures. Based on the systematic review, these policies typically involve price mark-up control measures to achieve their goals. The major impact of these control measures is improved affordability. By managing the drug prices, pharmaceutical expenditures can be optimised. This review could serve as a useful reference for health regulatory agencies in drafting cost control measures because it provided a detailed review of implementation of the price control policies, procedures and price mark-up control measures with regard to its success and challenges.	21
**FUNDING**			
Funding	27	This systematic review received no funding	

From: Moher D, Liberati A, Tetzlaff J, Altman DG, The PRISMA Group (2009). Preferred Reporting Items for Systematic Reviews and Meta-Analyses: The PRISMA Statement. PLoS Med 6(6): e1000097. doi:10.1371/journal.pmed1000097

Article Title: A Ssystematic Rreview of Ppharmaceutical price Mmark-up Ppractice and Iits Iimplementation

Journal: Exploratory Research in Clinical and Social Pharmacy

Article number: 100020

https://doi.org/10.1016/j.rcsop.2021.100020

References

1. Lee KS, Shahidullah A, Zaidi STR, Patel RP, Ming LC, Tariq MH, et al. The Crux of the Medicine Prices' Controversy in Pakistan. Front Pharmacol. 2017;8:504. Epub 2017/08/22. doi: 10.3389/fphar.2017.00504. PubMed PMID: 28824429; PubMed Central PMCID: PMCPMC5539127.
2. Ball D. Working Paper 3: The Regulation of Mark-ups in the Pharmaceutical Supply Chain. Review Series on Pharmaceutical Pricing Policies and Interventions. 2011.
3. Abdel Rida N, Mohamed Ibrahim MI, Babar Z-U-D, Owusu Y. A systematic review of pharmaceutical pricing policies in developing countries. J Pharm Health Serv Res. 2017;8(4):213-26. doi: 10.1111/jphs.12191.
4. Jakab I, Nemeth B, Elezbawy B, Karadayi MA, Tozan H, Aydin S, et al. Potential Criteria for Frameworks to Support the Evaluation of Innovative Medicines in Upper Middle-Income Countries-A Systematic Literature Review on Value Frameworks and Multi-Criteria Decision Analyses. Front Pharmacol. 2020;11:1203. Epub 2020/09/15. doi: 10.3389/fphar.2020.01203. PubMed PMID: 32922287; PubMed Central PMCID: PMCPMC7456841.
5. Franzen N, Retel VP, Schats W, van Harten WH. Evidence Underlying Policy Proposals for Sustainable Anticancer Drug Prices: A Systematic Review. JAMA Oncol. 2020;6(6):909-16. Epub 2020/03/28. doi: 10.1001/jamaoncol.2019.6846. PubMed PMID: 32215592.
6. Wettstein DJ, Boes S. Effectiveness of National Pricing Policies for Patent-Protected Pharmaceuticals in the OECD: A Systematic Literature Review. Appl Health Econ Health Policy. 2019;17(2):143-62. Epub 2018/10/28. doi: 10.1007/s40258-018-0437-z. PubMed PMID: 30367350.
7. Angelis A, Lange A, Kanavos P. Using health technology assessment to assess the value of new medicines: results of a systematic review and expert consultation across eight European countries. Eur J Health Econ. 2018;19(1):123-52. Epub 2017/03/18. doi: 10.1007/s10198-017-0871-0. PubMed PMID: 28303438; PubMed Central PMCID: PMCPMC5773640.
8. Md Hamzah N, Perera P, Rannan-Eliya R. How well does Malaysia achieve value for money in public sector purchasing of medicines? Evidence from medicines procurement prices from 2010 to 2014. BMC Health Serv Res. 2020;20. doi: 10.1186/s12913-020-05362-8.
9. Saeed A, Saeed H, Saleem Z, Fang Y, Babar Z-U-D. Evaluation of prices, availability and affordability of essential medicines in Lahore Division, Pakistan: A cross-sectional survey using WHO/HAI methodology. PLoS One. 2019;14(4):e0216122. doi: 10.1371/journal.pone.0216122.
10. Dave CV, Pawar A, Fox ER, Brill G, Kesselheim AS. Predictors of Drug Shortages and Association with Generic Drug Prices: A Retrospective Cohort Study. Value Health. 2018;21(11):1286-90. doi: 10.1016/j.jval.2018.04.1826.

11. Sarpatwari A, DiBello J, Zakarian M, Najafzadeh M, Kesselheim AS. Competition and price among brand-name drugs in the same class: A systematic review of the evidence. PLoS Med. 2019;16(7):e1002872. Epub 2019/07/31. doi: 10.1371/journal.pmed.1002872. PubMed PMID: 31361747; PubMed Central PMCID: PMCPMC6667132 following competing interests: ASK is a member of the Editorial Board of PLOS Medicine.
12. Vogler S, Schneider P, Lepuschutz L. Impact of changes in the methodology of external price referencing on medicine prices: discrete-event simulation. Cost Eff Resour Alloc. 2020;18(1):51. Epub 2020/12/10. doi: 10.1186/s12962-020-00247-3. PubMed PMID: 33292293; PubMed Central PMCID: PMCPMC7670789.
13. Vogler S, Schneider P, Zimmermann N. Evolution of Average European Medicine Prices: Implications for the Methodology of External Price Referencing. Pharmacoecon Open. 2019;3(3):303-9. Epub 2019/02/06. doi: 10.1007/s41669-019-0120-9. PubMed PMID: 30721410; PubMed Central PMCID: PMCPMC6710305.
14. Shaw B, Mestre-Ferrandiz J. Talkin' About a Resolution: Issues in the Push for Greater Transparency of Medicine Prices. Pharmacoeconomics. 2020;38(2):125-34. Epub 2020/01/21. doi: 10.1007/s40273-019-00877-3. PubMed PMID: 31956967.
15. Kanavos P, Fontrier A-M, Gill J, Efthymiadou O. Does external reference pricing deliver what it promises? Evidence on its impact at national level. Eur J Health Econ. 2020;21(1):129-51. doi: 10.1007/s10198-019-01116-4.
16. WHO. WHO guideline on country pharmaceutical pricing policies: World Health Organization; 2015.
17. Izadi E, Afshan G, Patel RP, Rao VM, Liew KB, Meor Mohd Affandi MMR, et al. Levofloxacin: Insights Into Antibiotic Resistance and Product Quality. Front Pharmacol. 2019;10:881. Epub 2019/09/03. doi: 10.3389/fphar.2019.00881. PubMed PMID: 31474853; PubMed Central PMCID: PMCPMC6702332.
18. Sharma D, Patel RP, Zaidi STR, Sarker MMR, Lean QY, Ming LC. Interplay of the Quality of Ciprofloxacin and Antibiotic Resistance in Developing Countries. Front Pharmacol. 2017;8:546. Epub 2017/09/06. doi: 10.3389/fphar.2017.00546. PubMed PMID: 28871228; PubMed Central PMCID: PMCPMC5566961.
19. Panteli D, Arickx F, Cleemput I, Dedet G, Eckhardt H, Fogarty E, et al. Pharmaceutical regulation in 15 European countries review. Health Syst Transit. 2016;18(5):1-122. Epub 2016/12/09. PubMed PMID: 27929376.
